# Autistic traits and mental health support service use among parents of young children with autism

**DOI:** 10.1016/j.rin.2026.100057

**Published:** 2026-08-22

**Authors:** Margaret G. Janse van Rensburg, Robert Hock, Emmanuella Asabea Twum, Resia-Maria Cooper, Abigail K. Reza Jameson, C. Andrew Conway, Mark E. Feinberg

**Affiliations:** a College of Social Work, University of South Carolina, Columbia, SC, United States; b The Pennsylvania State University, United States

**Keywords:** Autism spectrum disorder, Autistic traits, RAADS-14, Help-seeking, Caregiver support, Service use

## Abstract

Parents of children recently diagnosed with autism often experience elevated psychological distress, yet little is known about whether parental autistic traits are associated with support-seeking. Using baseline data from a randomized controlled trial of a parent mentoring intervention, this study examined support use among 355 parents of children aged 18 months to 8 years whose child had received an autism diagnosis within the previous 12 months. Parental autistic traits were assessed using the Ritvo Autism and Asperger Diagnostic Scale–14 (RAADS-14). Overall, 36.1% of parents reported accessing support for stress, emotional, or mental health concerns, and 33.2% scored above the RAADS-14 cutoff for elevated autistic traits. Parents with elevated autistic traits were more likely to report support use than those without elevated traits (46.6% vs. 30.8%). Elevated autistic traits were associated with greater odds of support use in both binary and continuous logistic regression models, and these associations remained significant after adjustment for demographic characteristics. However, associations were no longer statistically significant after accounting for anxiety and depressive symptoms. Anxiety symptoms emerged as the strongest independent predictor of support-seeking, whereas depressive symptoms were not significantly associated with support use. Among parents who accessed support, those with elevated autistic traits were significantly more likely to report using medical or psychiatric services. In exploratory analyses, fathers were less likely than mothers to access support overall, and anxiety symptoms appeared to be more strongly associated with support-seeking among fathers, although this finding should be interpreted cautiously. Findings suggest that elevated autistic traits are associated with differences in support use and service pathways, but that these associations are closely linked to co-occurring anxiety symptoms.

## Introduction

Parents of children with autism^1^ frequently report substantial psychological strain while navigating fragmented and often under-resourced systems of care, and these circumstances shape both their support needs and their help-seeking behaviour (Keenan et al., 2016). Across studies, parents describe complex and often prolonged pathways to diagnosis and support, with help-seeking unfolding through phases in which family members, professionals, schools, associations, and online communities can either facilitate access or create additional delays (Courcy & Riviéres-Pigeon, 2020; Rahman et al., 2023). Many parents report feeling responsible not only for coordinating care but also for locating appropriate services, often describing the process as having to “fight” the system to secure timely and suitable services for their children (Courcy & Riviéres-Pigeon, 2020; Radev et al., 2023). At the same time, parents identify a range of unmet needs for themselves, including clearer information, counselling, respite, and autism-informed services, alongside non-judgmental and culturally responsive support from professionals (Alibekova et al., 2022; Galpin et al., 2018; Huyen et al., 2025; Nayyef et al., 2025). Peer and parent-to-parent support can also be important, particularly when formal systems fall short, although such provisions are not experienced as supportive by all families (Bogush and Koronthály, 2025; Lee et al., 2023).

These support needs are particularly important in light of the elevated psychological distress experienced by many parents of autistic children. Research consistently shows that parents of autistic children experience higher levels of anxiety, stress, and depression than parents of typically developing children, and often compared with parents of children with other conditions (Almansour et al., 2013; Bitsika & Sharpley, 2004; Lam et al., 2024; Wang et al., 2021). Anxiety and depression are associated with poorer wellbeing and greater caregiver burden, underscoring the importance of ensuring that parents can access appropriate mental health support (Alicka et al., 2025; Almansour et al., 2013; Chen et al., 2024; Cohrs & Leslie, 2017; Kütük et al., 2021; Lam et al., 2024; Li et al., 2022). Understanding who accesses support and what factors facilitate or hinder that access is therefore of considerable importance for both research and practice.

One group that may be especially relevant in this context is parents who themselves show elevated autistic traits, often described as within the broader autism phenotype (BAP) (Gerdts & Bernier, 2011). A review of studies found BAP prevalence ranging from 2.6% to 80% among parents of autistic children, with higher rates among fathers than mothers (Rubenstein & Chawla, 2018). Parents with elevated autistic traits may face additional barriers to accessing healthcare and mental health support. Research indicates that many autistic adults report difficulty visiting a general practitioner when needed, compared with non-autistic adults, and commonly describe barriers such as stigma, sensory processing challenges in waiting rooms, difficulties planning and organizing for healthcare appointments, and communication barriers with providers (M. Doherty et al., 2021, 2022). Stigma and stereotyping by healthcare providers have been identified as particularly significant, driving avoidance and distrust of services (Mason et al., 2019). Beyond provider stigma, autistic adults often describe barriers related to communication differences, difficulties identifying and articulating support needs, concerns about being misunderstood, and challenges finding services that feel psychologically safe and autism-informed. Such experiences may contribute to avoidance of services and reduced help-seeking (Mason et al., 2019; Wilson et al., 2025). At the same time, anxiety is highly common among autistic people and depression is also elevated across the lifespan, suggesting that parents with elevated autistic traits may be navigating service access while also experiencing greater emotional distress (Hollocks et al., 2019; Hudson et al., 2018; Lai et al., 2019; Van Steensel et al., 2011). Consequently, parents with elevated autistic traits may represent a subgroup facing both the challenges associated with raising an autistic child and an increased vulnerability to anxiety and depression themselves. Given these barriers, it remains unclear whether elevated autistic traits are associated with reduced support use, increased support use, or differences in the types of services accessed.

Demographic characteristics such as gender, age, race/ethnicity, and education are also consistently associated with differences in mental health service use and are therefore important to account for when examining service access (Berk et al., 2023; Cairney et al., 2014; Cook et al., 2017; Davies et al., 2004; Elshaikh et al., 2023; Mackenzie et al., 2006; Maiuolo et al., 2019; Schonbrun & Whisman, 2010; Sheehan et al., 2024; Smith & Hebdon, 2024; Susukida et al., 2015; Teo et al., 2022; Wade et al., 2023). Accordingly, these factors were considered when examining the association between parental autistic traits and support use.

This study addresses a notable gap in the literature by examining patterns of support use among parents of young children recently diagnosed with autism. Drawing on baseline data from 355 parents enrolled in the Autism Parent Navigators (APN) randomized controlled trial, we examined whether parental autistic traits were associated with support use, whether these associations remained after accounting for demographic characteristics, anxiety symptoms, and depressive symptoms, and whether parents with elevated autistic traits differed in the types of supports they accessed. In doing so, the study contributes to a more nuanced understanding of how parental autistic traits, anxiety, and depression may shape engagement with services.

## Data and methods

### Data

Data for this study were drawn from a randomized controlled trial evaluating the APN, a peer mentoring program for co-caregivers of autistic children. Recruitment began in 2020 and occurred over 40 months. Families were recruited through pediatric provider referrals, schools, social media, direct mailings, and outreach/tabling at community events.

Families were eligible if they had a child aged 18 months to 8 years who was diagnosed with autism within the prior 12 months, the child was the first in the family to receive a diagnosis of autism, caregivers were not already receiving parent mentoring services, and caregivers were currently engaged in a coparenting relationship (e.g., married or partnered couples, same-sex partners, dating couples, adoptive parents, step-parent families, or parent–grandparent dyads). Two caregivers per family were required to participate in study procedures. Assessments were conducted at baseline, post-intervention, and 12-month follow-up.

The present study drew on baseline data collected from 381 parents of autistic children who were enrolled in the APN study between 2020 and 2024. Analyses for the current study were restricted to participants with complete data on all variables included in the regression models, resulting in a final analytic sample of 355 parents. Parents/caregivers completed a comprehensive online baseline questionnaire containing more than 700 study variables across multiple domains, including parent demographics, education, employment, physical and mental health, service use, social support, caregiving strain, coparenting, family functioning, parenting practices, and child developmental and behavioral characteristics. Most measures were completed by both participating caregivers, although some child-focused assessments were completed by only one parent per family. Study procedures were approved by the University of South Carolina IRB, and all participants provided informed consent electronically prior to completing the baseline survey.

### Measures

The dependent variable was parent service use for stress, emotional, or mental health concerns. At baseline, parents were asked whether they had received any help in the past year for stress, emotional, or mental health problems, including counseling, medical services, group therapy, or other sources of support. Responses were coded as a binary variable indicating any service use (0 = no, 1 = yes). Parents were also asked about specific types of support accessed, including medical doctors or psychiatrists (combined as a single response option in the original questionnaire), counselors/psychologists/social workers, group therapy with a trained counselor, self-help or peer-led support groups, and clergy or faith-based support. Although parents reported the frequency of service use, each service type was recoded as a binary indicator (0 = no, 1 = yes) for analysis.

Parental autistic traits were assessed using the Ritvo Autism and Asperger Diagnostic Scale–14 (RAADS–14), a brief self-report screener designed to identify elevated autistic traits in adults. Following established scoring conventions (Eriksson et al., 2013), a cutoff score of 14 or higher was used to indicate elevated autistic traits. A binary variable was created (0 = RAADS–14 < 14, 1 = RAADS–14 ≥ 14) for group comparisons and logistic regression analyses. In addition, the continuous RAADS–14 total score was retained and examined in regression models to assess the robustness of findings across operationalizations of autistic traits. The RAADS–14 is a screening instrument and is not intended for diagnostic purposes but has been used in prior research to identify individuals with elevated autistic traits for group comparison. For example, Crompton et al. (2025) applied a comparable approach, using the RAADS–14 to identify elevated autistic traits and exclude non-autistic participants scoring above the threshold, supporting the use of this cutoff for distinguishing groups based on trait levels rather than formal diagnosis.

Parental mental health was assessed using measures of anxiety and depressive symptoms. Anxiety symptoms were assessed using an abbreviated eight-item measure derived from the Spielberger State-Trait Anxiety Inventory (STAI). The study questionnaire included eight anxiety items assessing symptoms experienced during the past seven days (e.g., feeling nervous, panic, worry, tension, and difficulty calming down), rated on a five-point scale ranging from 1 (Never) to 5 (Always). A mean anxiety score was calculated for participants who completed at least seven of the eight items, with higher scores indicating greater anxiety symptoms. In the present sample, the abbreviated measure demonstrated excellent internal consistency (α = .94).

Depressive symptoms were assessed using the 20-item Center for Epidemiological Studies Depression Scale (CES-D; Radloff, 1977). Participants reported the frequency of depressive symptoms experienced during the past week on a 4-point scale ranging from 0 (Rarely or none of the time) to 3 (Most or all of the time). Four positively worded items were reverse coded prior to scoring. A mean CES-D score was calculated for participants who completed at least 16 of the 20 items, with higher scores reflecting greater depressive symptomatology. Previous analyses of the study dataset indicated excellent internal consistency for the CES-D (α = .91).

Logistic regression models were used to examine whether parental autistic traits were associated with service use. Covariates were selected *a priori* based on their conceptual relevance to help-seeking behaviour, as identified in prior research. Demographic covariates were obtained through participant self-report and included parent age (continuous), parent gender (male vs. female), and race (White vs. non-White), which were retained across models to account for well-documented demographic differences in access to and utilization of mental health services (Cook et al., 2017; Elshaikh et al., 2023; Mackenzie et al., 2006; Sheehan et al., 2024; Susukida et al., 2015; Teo et al., 2022). Ethnicity was not included due to overlap with race and small cell sizes, which posed a risk of collinearity and unstable estimates. To examine whether associations differed by gender, exploratory interaction models were estimated. In addition to testing whether the association between parental autistic traits and support use differed by gender, supplementary analyses examined whether the associations of anxiety and depressive symptoms with support use varied by parent gender.

Additional variables reflecting socioeconomic resources (education), family context (partner status, coparenting quality, and child intellectual or learning disability), and parent health were examined in preliminary analyses, given evidence linking these factors to caregiver stress, service need, and healthcare access (Cairney et al., 2014; Feng & Teti, 2025; Guerra et al., 2025; Hertveldt et al., 2025; Jansen et al., 2018; Langellier et al., 2020; Oliveira et al., 2025; Schonbrun & Whisman, 2010). However, these variables were not retained in final models as they did not meaningfully improve model fit or alter the association between parental autistic traits and service use. Analyses were limited to participants with complete data on all variables included in the regression models, resulting in the exclusion of 26 cases.

### Analysis

All analyses were conducted using SPSS for Mac, and no weighting procedures were required. Analyses proceeded in three stages. First, descriptive statistics were used to summarize sample characteristics and patterns of support access. Bivariate associations between parental autistic trait status and support use were examined using chi-square (χ^2^) tests. These analyses assessed both overall support access and differences across specific types of services. For statistically significant associations, effect sizes were reported using Cohen’s phi (φ), and odds ratios (ORs) with 95% confidence intervals were calculated to estimate the relative likelihood of service use among parents with elevated autistic traits compared to those without elevated autistic traits.

Following bivariate analyses, binary logistic regression models were estimated to examine whether parental autistic traits independently predicted support access. Parental autistic traits were modeled both as a binary indicator (RAADS–14 ≥ 14) and as a continuous measure (RAADS–14 total score) to assess the robustness of findings across operationalizations. Adjusted models included core demographic covariates (parent age, gender, and race) selected a priori based on their relevance to help-seeking behavior. A supplementary model additionally included depressive symptoms and anxiety symptoms to examine whether associations between autistic traits and support access were independent of parent mental health. Standard logistic regression was used because the outcome and predictor variables were measured at the individual parent level, with the individual parent as the unit of analysis. The potential non-independence of observations among coparenting adults is acknowledged as a study limitation.

To examine whether associations differed by gender, an interaction term between parental autistic traits and gender was tested in exploratory logistic regression models. Model fit was evaluated using −2 log likelihood, Cox and Snell R^2^, Nagelkerke R^2^, and the Hosmer–Lemeshow goodness-of-fit test.

## Results

### Sample characteristics

A total of 355 parents were included in the analytic sample. Parents ranged in age from 22 to 64 years (M = 36.71, SD = 6.88). The majority of participants self-identified as White/Caucasian (64.2%, n = 228), with 27.9% (n = 99) identifying as Black/African American and smaller proportions identifying as Asian or Pacific Islander, another race, or more than one race. Most parents identified as not Hispanic (92.7%, n = 329). Just over half of the sample identified as female (54.1%, n = 192). Educational attainment was evenly split, with 50.1% (n = 178) reporting a bachelor’s degree or higher. Nearly all participants reported that a spouse or romantic partner lived in the home (95.5%, n = 339). With respect to self-rated health, most parents described their overall health as good or excellent (75.8%, n = 269). Parents reported relatively low levels of depressive symptoms (CES-D: M = 0.69, SD = 0.53) and anxiety symptoms (STAI anxiety score: M = 2.05, SD = 0.93). Full demographic characteristics are presented in Table 1.

### Bivariate statistical results (Tables 2 and 3)

Overall, 128/355 (36.1%) parents reported accessing at least one support in the prior year. Parents with elevated autistic traits (i.e., RAADS–14 ≥ 14; n = 118) were more likely to report any support use than parents without elevated autistic traits (46.6% vs. 30.8%), χ^2^(1, N = 355) = 8.54, p = .003, φ = .16.

When examining specific types of support among parents who accessed any services (n = 128), a significant difference emerged for use of medical doctors or psychiatrists. Parents with elevated autistic traits were significantly more likely to report accessing a medical doctor or psychiatrist (85.5%, n = 47) compared to those without elevated autistic traits (60.3%, n = 44). This difference was statistically significant, χ^2^(1, N = 128) = 9.68, p = .002, with a moderate effect size (φ = .28). Parents with elevated autistic traits had nearly four times the odds of accessing a medical doctor or psychiatrist compared to those without elevated autistic traits (OR = 3.87, 95% CI [1.60, 9.37]). No other statistically significant differences were observed across support types, suggesting that differences between groups may be more specific to formal medical or psychiatric service use.

### Logistic regression results (Tables 4 and 5)

Logistic regression analyses indicated that parental autistic traits were significantly associated with support access. When modeled as a binary variable (RAADS–14 ≥ 14), parents with elevated autistic traits had nearly twice the odds of accessing support compared to parents without elevated autistic traits (OR = 1.96, 95% CI [1.25, 3.09], p = .003; Table 4). This association remained statistically significant after adjusting for parent age, gender, and race (OR = 1.86, 95% CI [1.15, 3.01], p = .012).

Similar findings were observed when parental autistic traits were modeled continuously (Table 5). Higher RAADS–14 scores were associated with greater odds of support use (OR = 1.04, 95% CI [1.01, 1.06], p = .002). This association remained statistically significant after adjusting for parent age, gender, and race (OR = 1.03, 95% CI [1.01, 1.06], p = .013), indicating that findings were consistent across operationalizations of autistic traits.

Across adjusted models, parent gender was a significant independent predictor of support access, with male parents showing substantially lower odds of service use than female parents (ORs = 0.33–0.47, ps≤.003). Parent age and race were not significantly associated with support access.

To examine whether the association between parental autistic traits and support use was independent of broader mental health symptoms, depressive symptoms and anxiety symptoms were added to the models. In both the binary and continuous RAADS models, the association between autistic traits and support use was no longer statistically significant after adjustment for mental health symptoms (binary model: OR = 0.93, 95% CI [0.53, 1.63], p = .804; continuous model: OR = 0.99, 95% CI [0.96, 1.02], p = .327). Anxiety symptoms were independently associated with greater odds of service use in both models (ORs = 2.44–2.59, ps <.001), whereas depressive symptoms were not significantly associated with support access (ORs = 1.11–1.12, ps ≥.756).

Model fit improved after the addition of anxiety and depressive symptoms, with Nagelkerke R^2^ increasing from.121 in the demographic-adjusted RAADS model to.256–.258 in the models that included mental health symptoms. In these fully adjusted models, the association between parental autistic traits and support use was no longer statistically significant, while anxiety symptoms remained a significant predictor of support use and depressive symptoms did not.

To examine whether the association between RAADS–14 scores and support use differed by gender, an interaction term between RAADS–14 scores and gender was tested. The interaction was not statistically significant (p = .642), indicating that the association did not differ by gender.

As an exploratory analysis, we also examined whether associations between mental health symptoms and support use differed by parent gender (Table 6). The anxiety × gender interaction was statistically significant, indicating that the positive association between anxiety symptoms and support use was stronger among fathers than mothers. In contrast, the depression × gender interaction was not significant. Although fathers were less likely overall to report support use, the association between anxiety symptoms and support-seeking was stronger among fathers than among mothers.

Additional covariates (e.g., education, partner status, parent health, and child disability status) were tested but not retained in final models, as they did not improve model fit or alter the association between parental autistic traits and support use.

## Discussion

This study examined whether parents of children recently diagnosed with autism who had elevated autistic traits differed in their patterns of support access. Initial analyses indicated a consistent association between parental autistic traits and support use. Across bivariate analyses and both binary and continuous logistic regression models, parents with elevated autistic traits were more likely to report accessing support in the past year. Importantly, findings were consistent regardless of whether autistic traits were operationalized dichotomously or continuously, suggesting that the observed association was robust across different approaches to measuring autistic traits.

Among parents who accessed support, those with elevated autistic traits were substantially more likely to report accessing a medical doctor or psychiatrist. Differences across other support types were not statistically significant. This pattern is consistent with research suggesting that autistic individuals may prefer more structured and formalized systems of care, while facing barriers to engaging with less structured or socially mediated forms of support such as peer groups (Adams & Young, 2021; M. Doherty et al., 2022). It may also reflect the role of stigma and prior negative service experiences in shaping help-seeking pathways, with formal medical settings perceived as more predictable, credible, or legitimizing than informal support contexts (Mason et al., 2019; Schnyder et al., 2017). More broadly, this pattern is consistent with prior reports that parents of autistic children engage with services in heterogeneous ways, with considerable variability in both support uptake and the types of supports utilized (Ingersoll et al., 2011; Pohl et al., 2020).

The association between autistic traits and support use changed very little after adjusting for parent age, gender, and race, indicating that demographic differences were not responsible for the observed pattern. Parent gender, however, emerged as a robust independent predictor of support access across all adjusted models. Male parents were substantially less likely to report accessing support than female parents, even after accounting for autistic traits, anxiety symptoms, and depressive symptoms. This finding is consistent with documented lower rates of mental health help-seeking among men, which has been attributed to gendered norms surrounding emotional expression, self-reliance, and stigma associated with seeking psychological support (Mackenzie et al., 2006). In contrast, parent age and race were not significantly associated with support access in the present sample.

The most notable finding emerged when anxiety and depressive symptoms were added to the regression models. In both the binary and continuous RAADS models, the association between autistic traits and support use was no longer statistically significant after accounting for mental health symptoms. At the same time, anxiety symptoms were the strongest independent predictor of support access, whereas depressive symptoms were not significantly associated with service use. These findings indicate that the association between autistic traits and support access was no longer statistically significant after adjustment for anxiety and depressive symptoms, with anxiety symptoms emerging as the strongest independent predictor of support access.

Exploratory analyses further examined whether associations between mental health symptoms and support use differed by parent gender. Although fathers were less likely overall to access support than mothers, a significant anxiety × gender interaction indicated that the association between anxiety symptoms and support-seeking was stronger among fathers. In contrast, the depression × gender interaction was not statistically significant. This pattern suggests that while fathers generally reported lower rates of support use, increases in anxiety symptoms may be associated with a particularly strong increase in the likelihood of seeking support. One possible interpretation is that fathers may delay seeking support until symptoms become more severe, whereas mothers may access support across a broader range of distress levels. However, given the exploratory nature of these analyses, this finding should be interpreted cautiously and replicated in future research.

This interpretation is consistent with a substantial body of literature demonstrating elevated rates of anxiety among autistic individuals and those with elevated autistic traits. Anxiety is among the most common co-occurring mental health conditions in autism, with estimates suggesting that approximately 20–40% of autistic individuals meet criteria for an anxiety disorder and even higher proportions report clinically significant anxiety symptoms (Hollocks et al., 2019; Lai et al., 2019; Van Steensel et al., 2011). Compared with non-autistic populations, autistic individuals experience anxiety at rates that are two to three times higher, with particularly elevated rates of social anxiety, specific phobias, obsessive-compulsive symptoms, and anxiety related to uncertainty and change (Kirsch et al., 2019; Lai et al., 2019; Vasa et al., 2020). Within this context, elevated autistic traits may co-occur with greater anxiety symptoms, and both may be associated with increased support use. However, the cross-sectional design precludes conclusions regarding the direction or mechanisms underlying these associations.

Interestingly, depressive symptoms did not independently predict support use despite extensive evidence that depression is elevated among autistic individuals and parents of autistic children (Chen et al., 2024; Hollocks et al., 2019; Hudson et al., 2018). One possible explanation is that anxiety and depression may influence help-seeking in different ways. Anxiety is often characterized by heightened worry, vigilance, and efforts to reduce distress, which may increase motivation to seek professional support. In contrast, depression is frequently associated with low motivation, fatigue, hopelessness, and social withdrawal, factors that may reduce the likelihood of initiating or maintaining engagement with services. Although speculative, this may be one possible interpretation of why anxiety emerged as a strong predictor of support access whereas depressive symptoms did not. This finding is notable given the well-documented prevalence of depression among autistic individuals and parents of autistic children, and suggests that different forms of psychological distress may have distinct implications for help-seeking behaviour. While anxiety was associated with support use and depression was not, it does not mean depression is unimportant in this population.

More broadly, these findings suggest that elevated autistic traits may identify a subgroup of parents with elevated mental health needs and distinct patterns of mental health service engagement, rather than simply serving as an unmeasured background characteristic. At the same time, findings from the service-type analyses suggest that differences may exist not only in whether support is accessed, but also in the types of services that are used.

Several plausible mechanisms may underlie these findings, including greater perceived need, elevated anxiety symptoms, prior engagement with mental health services, preferences for structured or biomedical models of care, and barriers to accessing peer- or community-based supports. However, these mechanisms remain speculative and require direct investigation. Given the cross-sectional nature of the data, findings should be interpreted as indicating differences in the likelihood and type of support accessed rather than causal effects of elevated autistic traits on help-seeking behaviour.

The contribution of this work is twofold. First, the findings suggest that elevated autistic traits may identify a subgroup of parents with elevated anxiety symptoms and distinct patterns of service engagement in the context of a child’s recent autism diagnosis. Second, the findings have practical implications for service systems. If parents with elevated autistic traits are more likely to access support through medical and psychiatric pathways, these providers may represent an important point of contact for supporting parent wellbeing. As such, efforts to improve provider capacity to recognize caregiver stress, communicate in neuroinclusive ways, and provide appropriate referrals may be particularly important. Practical adaptations may include clearer and more direct communication, sensory-considerate environments, and flexible approaches to delivering parent-facing resources. Such efforts may help ensure that supports are accessible and responsive to the diverse needs of families of autistic children.

Taken together, the results suggest that elevated autistic traits in parents are associated with differences in support access and service pathways, although these associations appear to be closely linked to co-occurring anxiety symptoms. By documenting this pattern, the present study provides a foundation for developing more intentional and neuroinclusive supports for parents of children recently diagnosed with autism. Future research should extend this work using longitudinal designs to clarify directionality, examine other forms of neurodivergence beyond autistic traits (e.g., ADHD), compare screening-based and clinically diagnosed samples, and incorporate qualitative methods to better understand caregiver preferences, barriers to support access, and decision-making processes.

### Limitations

Several limitations merit attention. First, the cross-sectional design precludes causal inference about whether parental autistic traits influence help-seeking or whether engagement with services shapes self-reported traits or awareness. Longitudinal research is needed to clarify the directionality of these associations. Additionally, although children had received an autism diagnosis within the previous 12 months, parent service use was assessed over the preceding year, whereas anxiety and depressive symptoms reflected the past week. Consequently, the temporal ordering of diagnosis, support use, and current mental health symptoms cannot be established, and some reported service use may have occurred before the child’s diagnosis. Second, service use was self-reported and operationalized as a binary outcome, which does not capture variation in the frequency, intensity, or perceived helpfulness of support and may obscure more nuanced patterns of help-seeking. Furthermore, the original questionnaire combined medical doctors and psychiatrists into a single response category, precluding separate examination of these potentially distinct forms of service use and help-seeking pathways. Third, parental autistic traits were assessed using the RAADS–14, a brief screening instrument with acceptable sensitivity but only moderate specificity. As such, findings should be interpreted as associations with elevated autistic traits rather than clinically confirmed diagnoses. Relatedly, much of the literature used to contextualize these findings draws from studies of clinically diagnosed autistic adults rather than individuals with elevated autistic traits identified through screening measures. Accordingly, parallels between these populations should be interpreted cautiously, as the experiences and support needs of individuals with elevated autistic traits may not fully align with those of clinically diagnosed autistic adults. In addition, anxiety symptoms were assessed using an abbreviated 8-item version of the STAI rather than the full instrument. Although the abbreviated measure demonstrated excellent internal consistency, it differs from the full STAI and may not capture the full breadth of anxiety symptoms.

Fourth, although the analyses assume independence of observations, the study design required participation from coparenting adults, meaning that some respondents were drawn from the same family unit. In such cases, help-seeking behaviours may be partially shared or coordinated, resulting in non-independence of observations and potentially underestimated standard errors. The present analyses did not account for this clustering. Future research should explicitly account for family-level clustering (e.g., through multilevel modelling or generalized estimating equations) or examine whether findings are robust when analyses are restricted to one parent per family. Finally, the APN sample required parents to be living with another adult and willing to participate in an online intervention, which may limit generalizability to single caregivers or those with limited digital access. In addition, participation in a randomized trial may introduce selection bias, as families willing to enroll in an intervention study may differ systematically from those who do not.

### Conclusion

Among parents whose child had received an autism diagnosis within the previous 12 months, those with elevated autistic traits were initially more likely to report accessing support. However, these associations appeared to be closely linked to co-occurring anxiety symptoms, which emerged as the strongest independent predictor of support use. These findings suggest that variation in parental characteristics may shape not only whether support is accessed, but also how families engage with different service pathways. Recognizing this heterogeneity has important implications for the design of caregiver supports, highlighting the need for more flexible, accessible, and neuroinclusive service systems that can accommodate diverse mental health needs, preferences, and patterns of help-seeking.

## Figures and Tables

**Table 1:** Demographics and study variables (N = 355).

Variable	Categories or Range	%
RAADS–14 score (continuous)	Range = 0–40; M = 10.73 (SD = 9.57)	
Parent has elevated autistic traits (RAADS–14 ≥ 14)	No	66.8% (n = 237)
	Yes	33.2% (n = 118)
Mental health services/support accessed	No	63.9% (n = 227)
	Yes	36.1% (n = 128)
CES-D depressive symptoms score	Range = 0–3; M = 0.69 (SD = 0.53)	
STAI anxiety score	Range = 1–5; M = 2.05 (SD = 0.93)	
Age (years)	Range = 22–64; M = 36.71 (SD = 6.88)	
Race	White/Caucasian	64.2% (n = 228)
	Black/African American	27.9% (n = 99)
	Asian/Pacific Islander	2.0% (n = 7)
	Other	1.7% (n = 6)
	More than one race	4.2% (n = 15)
Ethnicity	Hispanic	7.3% (n = 26)
	Not Hispanic	92.7% (n = 329)
Gender	Female	54.1% (n = 192)
	Male	45.9% (n = 163)
Education	Less than high school	2.3% (n = 8)
	High school or equivalency	12.7% (n = 45)
	Vocational training	3.9% (n = 14)
	Some college	20.8% (n = 74)
	Associate’s degree	10.1% (n = 36)
	Bachelor’s degree	24.5% (n = 87)
	Graduate/professional degree	25.6% (n = 91)
Self-reported Health	Excellent	16.1% (n = 57)
	Good	59.7% (n = 212)
	Fair	22.3% (n = 79)
	Poor	2.0% (n = 7)
Child gender	Female	26.5% (n = 94)
	Male	73.5% (n = 261)
Child ID/LD	No	60.3% (n = 214)
	Yes	39.7% (n = 141)
Partner in home	No	4.5% (n = 16)
	Yes	95.5% (n = 339)

*Notes*. Percentages may not sum to 100 due to rounding. RAADS–14 = Ritvo Autism and Asperger Diagnostic Scale (14-item). CES-D = Center for Epidemiological Studies Depression Scale. STAI = State-Trait Anxiety Inventory. Graduate/professional degree includes master’s degrees, PhDs, law degrees (JD), and professional doctorates (e.g., MD, AuD, DPT).

**Table 2:** Association between elevated parental autistic traits and support access (N = 355).

Elevated Parental Autistic Traits	No support	Any support
RAADS < 14 (n = 237)	69.2% (n = 164)	30.8% (n = 73)
RAADS ≥ 14 (n = 118)	53.4% (n = 63)	46.6% (n = 55)

**Notes.** RAADS–14 = Ritvo Autism and Asperger Diagnostic Scale (14-item). χ^2^(1, N = 355) = 8.54, p = .003, φ = .16; OR = 1.96, 95% CI [1.25, 3.09].

**Table 3:** Types of support accessed by parents (N = 128).

Type of support	RAADS < 14 (n = 73),	RAADS ≥ 14 (n = 55),	χ^2^ (df)	p	Effect size
Medical doctor/psychiatrist	60.3% (n = 44)	85.5% (n = 47)	9.678 (1)	.002	φ = .275; OR = 3.87 (1.60–9.37)
Counselor/psychologist/social worker	58.9% (n = 43)	67.3% (n = 37)	0.937 (1)	.333	φ = .086; OR = 1.43 (0.69–2.98)
Group therapy (trained counselor)	9.6% (n = 7)	16.4% (n = 9)	1.316 (1)	.251	φ = .101; OR = 1.85 (0.64–5.31)
Self-help/peer group	6.8% (n = 5)	10.9% (n = 6)	0.658 (1)	.417	φ = .072; OR = 1.67 (0.48–5.77)
Clergy/ faith-based support	11% (n = 8)	5.5% (n = 3)	1.210 (1)	.271	φ = −.097; OR = 0.47 (0.12–1.86)

**Note.** OR = odds ratio. RAADS–14 = Ritvo Autism and Asperger Diagnostic Scale (14-item). N = 128 reflects the subset of participants who reported receiving help and completed the subsequent service-type items.

**Table 4:** Binary RAADS logistic regression predicting support access (N = 355).

Predictor	Model 1 Unadjusted OR (95% CI), p	Model 2 Demographics Adjusted OR (95% CI), p	Model 3 Mental Health Adjusted OR (95% CI), p
Parents with elevated autistic traits (RAADS ≥ 14)	1.96 (1.25–3.09),.003	1.86 (1.15–3.01),.012	0.93 (0.53–1.63),.804
Age (years)	—	0.99 (0.96–1.03),.614	0.99 (0.95–1.03),.482
Male (vs. female)	—	0.33 (0.21–0.53), < .001	0.46 (0.28–0.77),.003
White (vs. non-White)	—	1.28 (0.80–2.06),.308	1.07 (0.65–1.78),.782
CES-D mean depressive symptoms score			1.11 (0.56–2.20),.770
STAI anxiety score			2.44 (1.60–3.72), < .001
**Model Fit**			
−2 Log likelihood	455.737	431.287	390.915
Cox & Snell R^2^	.023	.088	.186
Nagelkerke R^2^	.032	.121	.256
Hosmer–Lemeshow (p)	—	.315	.298
N	355	355	355

**Note.** OR = odds ratio. RAADS–14 = Ritvo Autism and Asperger Diagnostic Scale (14-item). CES-D = Center for Epidemiological Studies Depression Scale. STAI = State-Trait Anxiety Inventory.

**Table 5:** Continuous RAADS logistic regression predicting support access (N = 355).

Predictor	Model 1 Unadjusted OR (95% CI), p	Model 2 Demographics Adjusted OR (95% CI), p	Model 3 Mental Health Adjusted OR (95% CI), p
RAADS–14 total score	1.04 (1.01–1.06),.002	1.03 (1.01–1.06),.013	0.99 (0.96–1.02),.327
Age (years)	—	0.99 (0.96–1.03),.594	0.98 (0.95–1.02),.405
Male (vs. female)	—	0.34 (0.21–0.54), < .001	0.47 (0.28–0.77),.003
White (vs. non-White)	—	1.28 (0.80–2.06),.308	1.07 (0.65–1.78),.787
CES-D mean depressive symptoms score			1.12 (0.56–2.22),.756
STAI anxiety score			2.59 (1.68–4.01), < .001
**Model Fit**			
−2 Log likelihood	454.526	431.377	390.005
Cox & Snell R^2^	0.027	0.088	0.189
Nagelkerke R^2^	0.037	0.121	0.258
Hosmer–Lemeshow (p)	0.685	0.597	0.089
N	355	355	355

**Note.** OR = odds ratio. RAADS–14 = Ritvo Autism and Asperger Diagnostic Scale (14-item). CES-D = Center for Epidemiological Studies Depression Scale. STAI = State-Trait Anxiety Inventory.

**Table 6:** Exploratory logistic regression examining gender moderation of anxiety and depression associations with support-seeking (N = 355).

Interaction	OR	95% CI	p
Depression × Gender	0.56	0.13, 2.42	.433
Anxiety × Gender	3.15	1.23, 8.10	.017

**Note.** OR = odds ratio. ORs are derived from a logistic regression model predicting support-seeking that included age, race, parent gender, elevated autistic traits, anxiety symptoms, depressive symptoms, and the corresponding interaction terms. Gender was coded 0 = female, 1 = male.

## Data Availability

Data are available upon reasonable request with ethical clearance and confidentiality agreements.

## Footnotes

1 In this article, we use both person-first (e.g., “children with autism”) and identity-first (e.g., “autistic children”) language. This approach reflects respect for the diversity of preferences within the autism community and acknowledges that no single terminology is universally accepted.
